# Unveiling the Molecular Mechanisms of Rosacea: Insights From Transcriptomics and In Vitro Experiments

**DOI:** 10.1111/jocd.16753

**Published:** 2025-01-16

**Authors:** Luzhu Chen, Juan Wang

**Affiliations:** ^1^ Department of Plastic and Cosmetic Surgery, Hubei Provincial Hospital of Traditional Chinese Medicine The Affiliated Hospital of Hubei University of Chinese Medicine Wuhan China

**Keywords:** HaCaT cells, inflammatory reaction, rosacea, toll‐like receptor, transcriptomics

## Abstract

**Background:**

Rosacea is a prevalent inflammatory skin condition, but its molecular mechanisms and treatment responses remain poorly understood.

**Aims:**

This study aims to investigate the molecular mechanisms underlying rosacea and explore drug response through transcriptomic analysis and in vitro experiments.

**Patients/Methods:**

We performed high‐throughput RNA sequencing to analyze gene expression patterns in rosacea patients. In vitro experiments, including RT‐qPCR, Western blot, ELISA, scratch, and Transwell assays, were used to evaluate gene and protein expression and cell behavior in HaCaT cells under simulated rosacea conditions.

**Results:**

Transcriptomic analysis revealed significantly elevated expression of inflammatory‐related genes in rosacea patients. In vitro, HaCaT cells exhibited enhanced proliferation and migration abilities, accompanied by increased expression of pro‐inflammatory genes and proteins. Specifically, Toll‐like receptor 2 (TLR2) and S100A9 proteins were upregulated, potentially promoting these processes.

**Conclusions:**

Our study elucidates the molecular mechanisms of rosacea, highlighting the role of inflammatory pathways and altered cell behavior in the disease. TLR2 and S100A9 may contribute to disease progression, offering potential targets for future therapeutic strategies.

## 1 Introduction

Rosacea is a common chronic skin disease characterized by facial skin erythema, papules, and pustules, severely impacting the patient's appearance and quality of life [1, 2, 3]. However, the pathogenesis of rosacea remains poorly understood at present [4, 5, 6]. Although some studies have identified potential influences of inflammatory reactions and genetic factors, there is still a lack of in‐depth understanding regarding its molecular mechanisms and individualized treatment strategies [7, 8, 9].

Transcriptomics, a high‐throughput technology for studying gene expression, has been widely applied to investigate the pathogenesis and treatment response of various diseases [10, 11, 12]. Therefore, in this study, we conducted a systematic analysis of gene expression patterns in rosacea patients using high‐throughput RNA sequencing technology. Through this approach, we can identify key genes and signaling pathways related to rosacea development and treatment response, providing important insights for a deeper understanding of the pathogenesis of this disease [13, 14, 15].

To investigate the molecular mechanisms and drug response of rosacea further, a series of in vitro experiments were performed. First, techniques such as RT‐qPCR, Western blot, and ELISA were used to assess the expression levels of rosacea‐related cytokines and key genes [16]. Additionally, scratch assays and Transwell experiments were employed to study cell migration and proliferation [17]. These experimental results provide important clues for a comprehensive understanding of the roles of inflammatory reactions and cellular functions in rosacea development.

The aim of this study is to reveal the pathogenesis and individualized treatment possibilities of rosacea by investigating its molecular mechanisms and drug response in depth. By utilizing a comprehensive research approach combining transcriptomics technology and in vitro experiments, we hope to gain a more comprehensive understanding of the development process of rosacea and explore new treatment strategies. The research findings may provide scientific evidence for individualized treatment and clinical practice, offering important information and directions to improve the quality of life of patients.

## 2 Materials and Methods

### 2.1 Analysis of Gene Expression Differences

In this study, we analyzed the rosacea dataset GSE65914 from the GEO database (https://www.ncbi.nlm.nih.gov/geo/) using the GPL570 platform. The dataset consists of 20 normal samples and 38 rosacea skin samples, with detailed information provided in Table S1. We set |log2FoldChange| > 1 and *p*‐value < 0.05 as significance thresholds and used the GeneCards database to obtain rosacea‐associated genes (Relevance score > 1). Subsequently, we utilized the “VennDiagram” package in R to generate a Venn diagram of the intersecting genes, thereby identifying differentially expressed genes in rosacea.

### 2.2 Enrichment Analysis and Construction of Protein Interaction Networks

To explore relevant molecular pathways, we performed GO and KEGG enrichment analysis on differentially expressed genes using the “clusterProfiler” package in R. During this process, we applied the Benjamini‐Hochberg multiple correction method with a *p*‐value cutoff of 0.05 and a *q*‐value cutoff of 0.2. Additionally, we conducted a protein functional network analysis using the STRING database (https://cn.string‐db.org/). To optimize the network visualization, we utilized cytoscape 3.9.1 and the “Analyze Network” feature to calculate the degree values of individual molecules.

### 2.3 Machine Learning‐Based Selection of Key Genes

We employed two machine learning methods, LASSO and Random Forest, for further selection of key genes related to rosacea. LASSO (least absolute shrinkage and selection operator) is a regression analysis method [18] that incorporates a penalty term on the coefficients to achieve feature selection and regularization, thereby enhancing the predictive accuracy and interpretability of the model. This method is particularly suitable for handling highly collinear or high‐dimensional data. Random Forest [19] is an ensemble learning algorithm that builds multiple decision trees and combines their results for prediction, making it applicable to both classification and regression tasks. Random Forest is capable of effectively dealing with large datasets and exhibits good robustness against missing and imbalanced data.

### 2.4 Cell Culturing and Treatment

In this experiment, human epidermal keratinocyte cells (HaCaT cells; catalog number T0020001; AddexBio, San Diego, California, USA) were cultured in a calcium‐free DMEM medium (Gibco, ThermoFisher Scientific, USA) containing 10% (v/v) FBS and 1% (w/v) penicillin–streptomycin. All cells were incubated at 37°C and 5% CO_2_ in a constant‐temperature incubator (Thermo Fisher Scientific, USA). To simulate rosacea, the cells were treated with LL‐37 (4 μM) (synthesized by Sangon Biological Technology, China, following the amino acid sequence LLGDFFRKSKEKIGKEFKRIVQRIKDFLRNLVPRTE, HPLC verified, purity over 95%) for 12 h, while the control group (NS group) was treated with an equivalent volume of sterile PBS [20].

For cell transfection, the viruses were purchased from Platinum Genomics (Shanghai, China). The sequences used were as follows: human sh‐NC: 5′‐CCTAAGGTTAAGTCGCCCTCG‐3′; human shTLR2 #1: GCAAGTTCCCTAATCACTTTA; human shTLR2 #2: GCTGATAGCATCTGGCCTTTA; shTLR2 #3: GGAATGAACCACATAGGAAGA; human shS100A9 #1: 5′‐GGGCCTGTTATGTCAAACTGT‐3′; human shS100A9 #2: 5′‐GGCCTGTTATGTCAAACTGTC‐3′; human shS100A9 #3: 5′‐GCCTGTTATGTCAAACTGTCT‐3′; mouse sh‐NC: 5′‐AATTCTCCGAACGTGTCACGT‐3′; mouse shTLR2: 5′‐TCTGCAAACTGCGCAAGATAA‐3′; mouse shS100A9: 5′‐GCTGAGCTTTGAGGAGTGTAT‐3′.

The lentiviral particles were packaged into HEK‐293 T cells (iCell‐h237, Cell Biotech Co. Ltd., Shanghai, China) using the lentivirus packaging kit (A35684CN, Invitrogen, USA). After 48 h, the cell supernatant was collected as the lentivirus, with a titer of 1 × 10^8^ TU/mL. The target cells were incubated with the lentivirus mixture at a 40% concentration for 8 h. Subsequently, the culture medium was replaced with DMEM containing 10% fetal bovine serum, and the cells were screened with 5 μg/mL puromycin (A1113803, Thermo Fisher Scientific, China) for 4 weeks before the experiments were conducted [21].

Cell groups were as follows: NS group (control cells); LL‐37 + shNC group (shNC lentivirus transfection + LL‐37 treatment); LL‐37 + shTLR2 group (shTLR2 lentivirus transfection + LL‐37 treatment); and LL‐37 + shS100A9 group (shS100A9 lentivirus transfection + LL‐37 treatment). Refer to Figure S1A for the detailed protocol of the in vitro cell experiment.

### 2.5 RT‐qPCR

Total RNA was extracted from tissues using Trizol (16 096 020, Invitrogen, USA). For mRNA detection, reverse transcription was performed using the reverse transcription kit (RR047A, Takara, Japan) to obtain cDNA. qRT‐PCR was conducted following the protocol provided in the TaqMan Gene Expression Assays protocol (Applied Biosystems, Foster City, CA, USA). GAPDH was used as the internal reference. The PCR program was set as follows: initial denaturation at 95°C for 10 min, followed by 35 cycles of denaturation at 95°C for 15 s, annealing at 60°C for 30 s, and extension at 72°C for 45 s. All qRT‐PCR reactions were performed in triplicate. The primer design is summarized in Table S2. The relative expression ratio between the experimental group and the control group was calculated using the 2^−ΔΔCt^ method, where ΔΔCT = ΔCt experimental group—ΔCt control group, and ΔCt = Ct target gene—Ct reference gene. Ct represents the cycle threshold at which the real‐time fluorescence intensity reaches the set threshold, corresponding to the logarithmic amplification phase [17]. The experiment was repeated three times.

### 2.6 Western Blot Detection

Cell pellets were transferred into 1.5 mL Eppendorf tubes, and 200 μL of RIPA lysis buffer (P0013B, Beyotime, Beijing, China) was added to each tube. After homogenization, the samples were placed on ice for 10 min, centrifuged (3000 *g*, 4°C, 10 min), and the supernatant was transferred to pre‐chilled Eppendorf tubes. The protein concentration was quantified using the BCA assay kit (A53226, Thermo Fisher Scientific, Rockford, IL, USA). After measuring the protein concentration with the BCA assay, the samples were adjusted with loading buffer and boiled (99°C, 10 min) and then stored at −20°C. The appropriate separation gel concentration was chosen based on the target protein molecular weight, with 20 μL loaded per well. The PVDF membrane was activated using methanol and transferred using a Bio‐Rad electrophoresis system adjusting the time according to protein molecular weight and using a fixed voltage or current. The membrane was blocked with 5% BSA at room temperature for 1 h, followed by incubation with the corresponding primary antibodies (human TLR2 (ab68159, 1:1000, Abcam, UK), human S100A9 (ab63818, 1:1000, Abcam, UK), human GAPDH (ab8245, 1:1000, Abcam, UK)) overnight at 4°C. The next day, the membrane was taken out and allowed to equilibrate at room temperature on an orbital shaker for 1 h, followed by three washes with TBST (containing 0.1% Tween 20) at room temperature. Then, a 1:20 000 diluted HRP‐conjugated goat anti‐rabbit IgG (ab205718, Abcam, UK) in TBST was added and incubated for 1 h at room temperature, followed by three additional washes with TBST. Finally, the membrane was visualized by adding the chemiluminescent substrate (P0019, Beyotime, Beijing, China) in a 1:1 ratio, evenly coating the membrane surface, and imaging with a gel imaging system. The protein bands were scanned for grayscale using AlphaView SA software (version: 3.4.0), and the relative protein content of the target protein was determined by dividing the grayscale value of the target protein band by that of the loading control GAPDH protein band [20, 22]. Each experiment was performed in triplicate.

### 2.7 Enzyme‐Linked Immunosorbent Assay (ELISA)

After collecting the cell culture supernatant, it was centrifuged at 1000 × *g* for 10 min at room temperature, and then the supernatant was collected. For tissue samples, cryopreservation was performed using isopentane. Briefly, 10 mg of tissue was homogenized in 2–3 mL of buffer, followed by centrifugation at 16 000*×g* for 30 min at 4°C to collect the supernatant for protein level analysis using the ELISA method. Strictly follow the instructions provided in the kit to determine the levels of IL‐6 (97068ES48, Yisheng Biotech Co. Ltd., Shanghai, China), OSM (HEO004, Shanghai Bogu Biotech Co. Ltd., Shanghai, China), and TNF‐α (97072ES48, Yisheng Biotech Co. Ltd., Shanghai, China) in the cell culture supernatant or tissue homogenate.

### 2.8 CCK‐8 Cell Proliferation Assay

The CCK‐8 assay kit was purchased from Boster Biological Technology (Wuhan, China). Cells were seeded in a 96‐well plate (Corning, USA) at a density of 1 × 10^3^ cells per well. After 24 h of culture, 10 μL of CCK‐8 reagent was added to each well containing 100 μL of culture medium, followed by incubation for an additional hour. Absorbance at the wavelength of 450 nm was measured using a microplate reader (BioTek, USA) to calculate the cell proliferation index [17].

### 2.9 Scratch Assay and Transwell Migration Assay

Cells (5 × 10^5^ per well) were seeded in a 6‐well plate. Once the cells reached 100% confluence, a scratch was created using a scalpel blade. Following the scratch, cells were washed with PBS and a medium containing 4 μM LL‐37, and an equal volume of PBS was added. After 24 h, the distance of the scratch was measured to assess cell migration capability.

Cell migration capability was evaluated using the Transwell system (CLS3398, Millipore, USA). Cells (1 × 10^4^) treated with PBS and LL‐37 (4 μM) for 12 h were seeded in the upper chamber of the Transwell, using 200 μL serum‐free medium for cultivation. The lower chamber was filled with medium containing 10% FBS. After 24 h of incubation, non‐migrating cells were removed, and migrating cells were fixed and stained with 1% crystal violet. Cell counting and analysis were performed using a microscope [17].

### 2.10 Collection of Human Skin Tissue Samples

All skin biopsy samples were collected from the central facial area of healthy subjects or rosacea patients (females aged 20–50) in the dermatology department of our hospital. A total of 8 female rosacea patients, diagnosed through clinical and pathological examinations, and 8 age‐matched healthy subjects were included in this study. The severity of rosacea was assessed using the CGS score. Information on the patients and healthy subjects is presented in Table S3. The use of all human samples was approved by the Ethics Committee of our hospital, with written informed consent obtained from all participants. The experiment complies with the principles outlined in the World Medical Association's Declaration of Helsinki [23, 24]. Portions of the collected skin tissue were fixed in 4% paraformaldehyde for immunohistochemical staining, while others were preserved in liquid nitrogen for RT‐qPCR and ELISA analysis.

### 2.11 Immunohistochemical Staining

The expression of TLR2 and S100A9 proteins in the skin tissue of rosacea patients was detected using the streptavidin peroxidase (SP) immunohistochemical method. Paraffin‐embedded skin tissue sections from rosacea patients were prepared, with consecutive sections (5 μm thick) subjected to routine dehydration and processed for immunohistochemical staining. Endogenous peroxidase activity was blocked by treating with 3% hydrogen peroxide at room temperature for 10 min, followed by blocking with normal goat serum for 10 min. Primary antibodies, anti‐human TLR2 (ab213676, 1:100, Abcam, UK) and anti‐human S100A9 (ab63818, 1:1000, Abcam, UK), were applied and incubated overnight at 4°C. After applying biotin‐labeled secondary antibody (goat anti‐rabbit, 1:500, BA1003, Boster, Wuhan, China) and incubating at 37°C for 20 min, 50 μL streptavidin‐biotin‐peroxidase solution was added and incubated at room temperature for 10 min. DAB was used for color development, followed by hematoxylin counterstaining, dehydration, clearing, and mounting for observation under a light microscope. Positive cells for protein expression were identified by brownish‐yellow staining, with the intensity of epidermal staining assessed on a scale of 0–4 (0 = none, 1 = weak/low, 2 = moderate, 3 = strong, 4 = very strong) [25, 26].

### 2.12 Statistical Analysis

Multiple statistical methods were employed in this study to ensure the accuracy and scientific validity of data analysis. Descriptive statistics were initially used to summarize sample characteristics, including mean, standard deviation, and frequency distribution. Transcriptomics data were evaluated for differences using ANOVA (analysis of variance) or independent sample *t*‐tests. Additionally, multivariate data were analyzed using principal component analysis (PCA) and cluster analysis to identify underlying patterns and associations. For mechanism validation, repeated measures ANOVA was used to analyze changes in time‐series data, such as the dynamic variations of drug efficacy. A statistical significance level of *p* < 0.05 was considered in all analyses. R language and specialized statistical software such as SPSS and GraphPad Prism were employed to implement these statistical methods. By employing this comprehensive statistical analysis approach, this study was able to thoroughly investigate the molecular pathological features and individualized drug response differences in rosacea.

## 3 Results

### 3.1 Analysis of Gene Expression Data Reveals 17 Upregulated Genes and 1 Downregulated Gene in Rosacea

**FIGURE 1:**
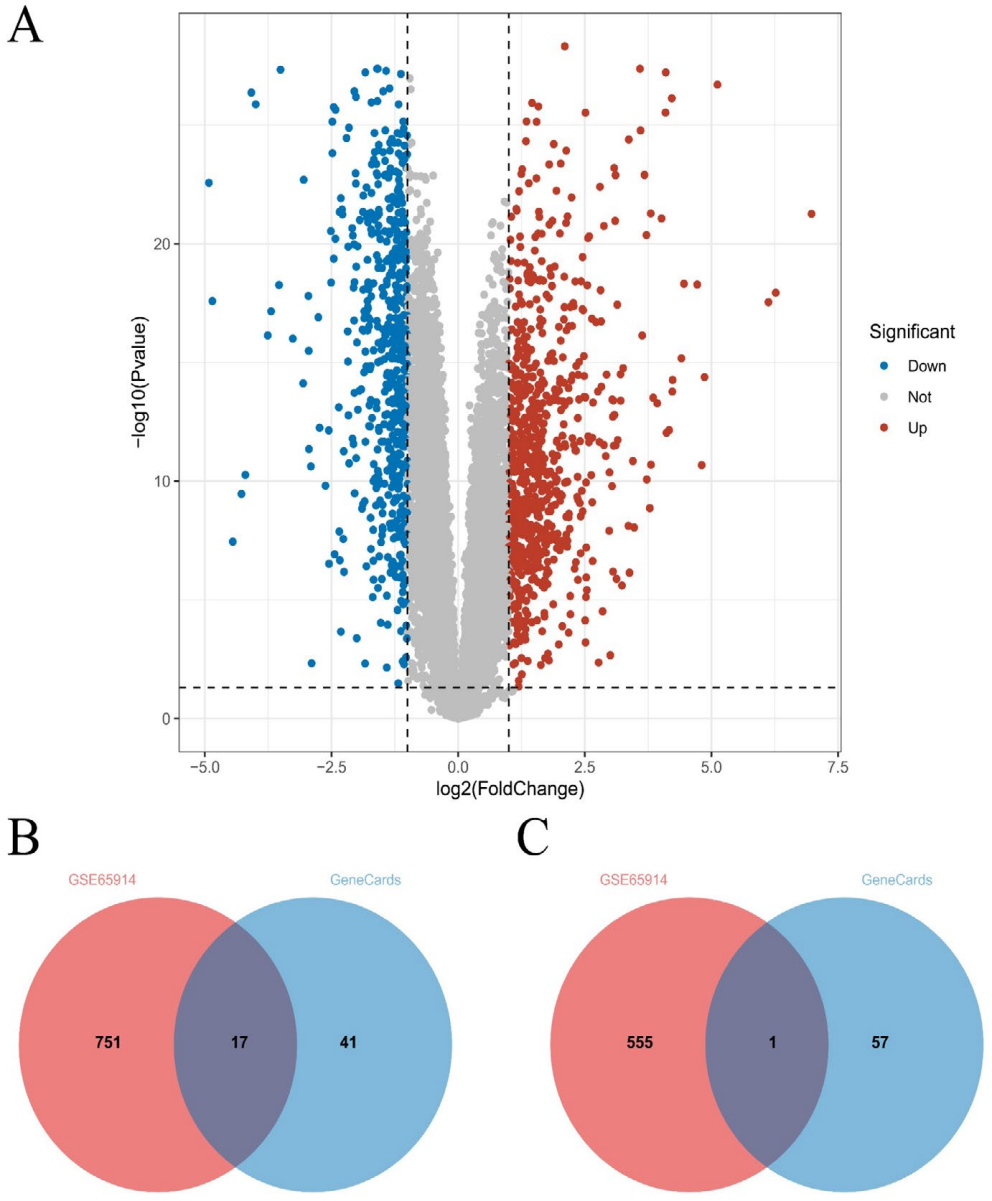
Differential expression genes in two different types of rosacea. (A) Volcano plot of differential expression genes in rosacea; (B) Venn diagram of the intersection between upregulated genes and rosacea‐related genes obtained from GeneCards; (C) Venn diagram of the intersection between downregulated genes and rosacea‐related genes obtained from GeneCards.

Rosacea is a common chronic skin disorder that primarily affects the facial area, particularly the nose, forehead, cheeks, and chin regions. Its characteristics include erythema, inflammation, telangiectasia (causing facial redness), and small pustules [1]. In‐depth research into the molecular mechanisms can help identify the underlying causes of this disease and develop more effective treatment methods. In this study, we performed differential gene expression analysis on the expression dataset GSE65914 for rosacea and identified 768 upregulated genes and 556 downregulated genes in rosacea (Figure 1A). After intersecting the differentially expressed genes with the rosacea‐related genes in GeneCards, we identified 17 upregulated genes (Figure 1B) and 1 downregulated gene (Figure 1C). The downregulated gene, XK, has been found in various tissues, particularly the brain, muscle, and heart, and is responsible for guiding protein synthesis [27]. However, the exact function of the XK protein is currently unclear. Therefore, in subsequent analyses, we will focus on these 17 upregulated genes.

### 3.2 Enrichment Analysis of Molecular Pathways and Key Genes in Rosacea

**FIGURE 2:**
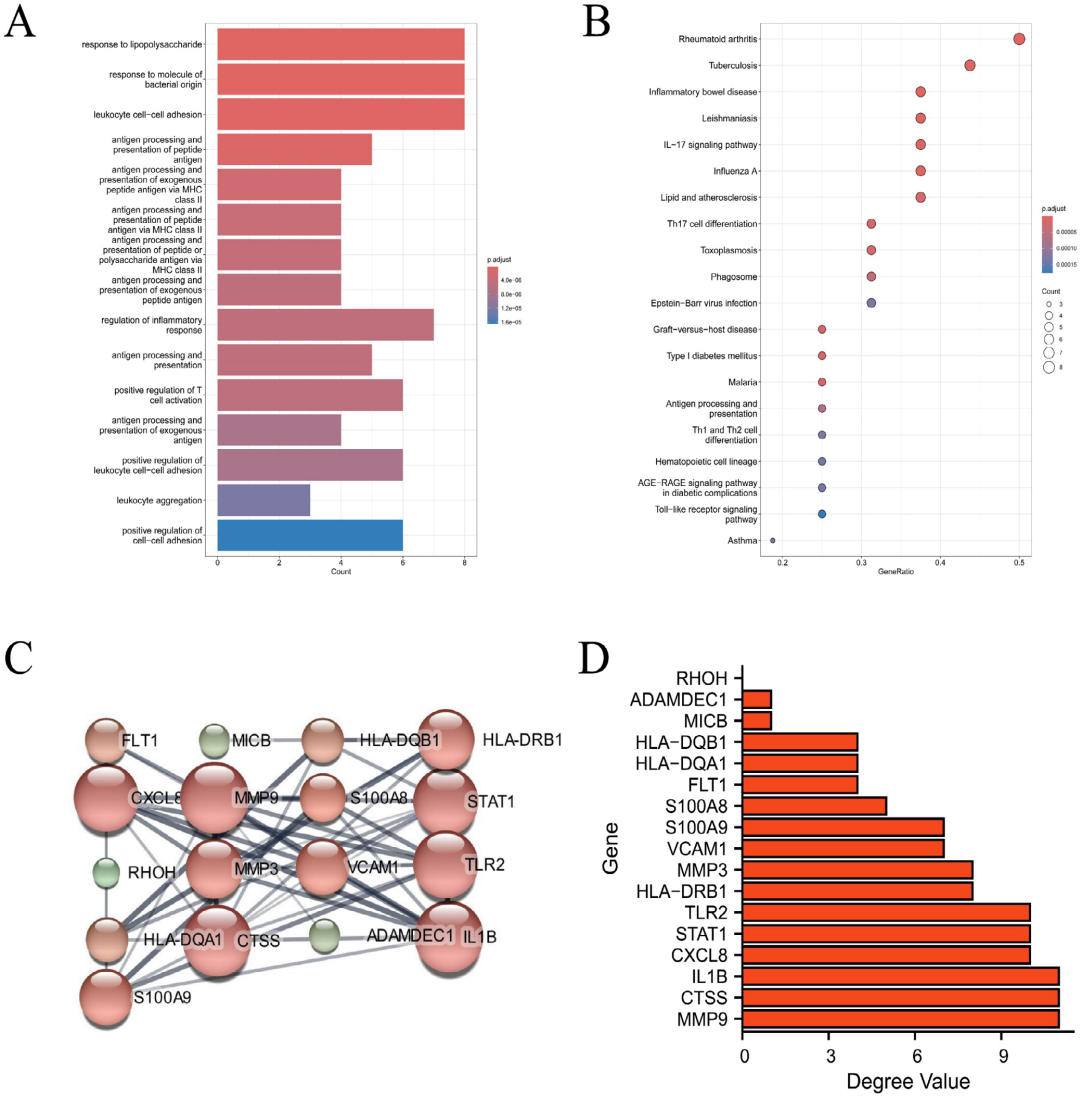
Enrichment analysis and PPI network construction of upregulated genes. (A) GO enrichment analysis results of upregulated genes in rosacea; (B) KEGG enrichment analysis results of upregulated genes in rosacea; (C) protein–protein interaction network construction of upregulated genes in rosacea; (D) degree values of each molecule.

To investigate the molecular pathways associated with upregulated genes in rosacea, we conducted enrichment analysis using GO and KEGG databases. The analysis revealed that upregulated genes in rosacea were significantly enriched in pathways related to lipopolysaccharide response, bacterial molecular response, antigen processing, and presentation, as well as leukocyte and inflammation‐related pathways in the GO analysis. In the KEGG analysis, enrichment was observed in pathways such as rheumatoid arthritis, tuberculosis, inflammatory bowel disease, and the IL‐17 signaling pathway. These pathways were highly associated with inflammation (Figure 2A,B), suggesting a crucial role of immune reactions related to inflammation in the development of rosacea. The control of inflammatory reactions is, therefore, important for the treatment of rosacea.

In the protein–protein interaction (PPI) network analysis, we calculated the degree values of 17 upregulated genes (Figure 2C,D). Degree values provide insights into the importance and centrality of each node in the network, whereas higher degree values usually indicate molecules that play critical roles in biological processes or disease states, such as key regulatory factors or potential drug targets [28]. Therefore, we filtered out molecules with degree values less than 3 and ultimately retained 14 genes for further analysis. These included HLA‐DQB1, GLA‐DQA1, FLT1, S100A8, S100A9, VCAM1, MMP3, HLA‐DRB1, TLR2, STAT1, CXCL8, IL1B, CTSS, and MMP9.

### 3.3 Identification of Key Genes Associated With Rosacea Through LASSO and Random Forest Algorithms: The Association Between S100A9 and TLR2

**FIGURE 3:**
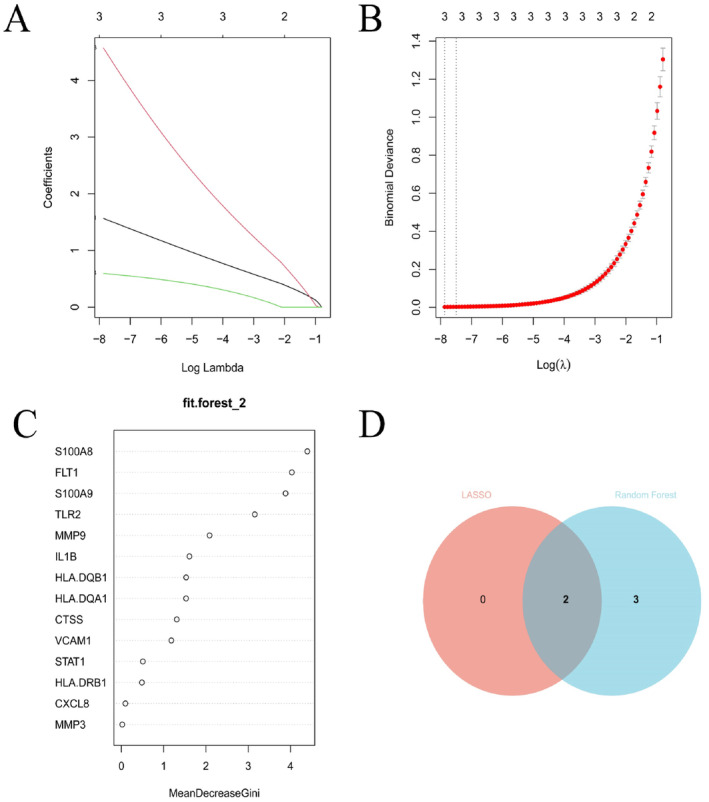
Machine learning selection of key genes. (A) LASSO coefficient path plot; (B) LASSO cross‐validation curve; (C) Random Forest ranking of importance of correlated molecules; (D) Venn diagram of the intersection between the two machine learning analysis results.

In this study, we employed two machine learning algorithms, LASSO and random forest, to further identify the pivotal genes involved in rosacea (Figure 3). In the LASSO analysis, a minimum *λ* value of 0.0004 was determined, resulting in the identification of three genes with nonzero coefficients. Moreover, through the random forest analysis, we found that the model yielded the lowest error rate when the number of variables included in the decision tree was four. Consequently, we constructed the model and ultimately included five genes, based on MeanDecreaseGini > 2, as key genes. The intersection of these two algorithms ultimately determined that S100A9 and TLR2 were the two critical molecules. S100A9, a protein belonging to the S100 protein family, plays a crucial role in regulating inflammation processes and immune responses, primarily through calcium signaling [29]. S100A9 typically forms a complex with S100A8 and is associated with various inflammatory diseases. TLR2, on the other hand, is an integral component of the immune system, primarily responsible for recognizing pathogen‐associated molecular patterns (PAMPs) and playing a critical role in innate immune responses, aiding the body in identifying and combating microbial infections [30]. The results obtained from the identification of these two key genes suggest a strong correlation between rosacea and inflammation.

### 3.4 Enhanced Proliferation and Migration Abilities of HaCaT Cells in a Rosacea Environment Through Regulation by TLR2 and S100A9

**FIGURE 4:**
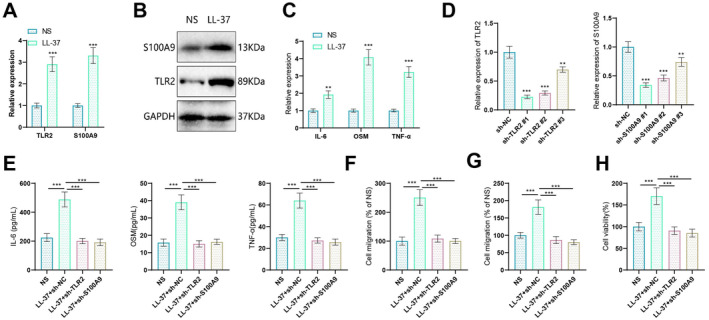
Effects of rosacea microenvironment on proliferation, inflammatory reaction, and migration of HaCaT cells in vitro. (A) RT‐qPCR detection of mRNA expression changes in TLR2 and S100A9 induced by LL‐37; comparison of proliferation rates of keratinocytes and fibroblasts in a simulated rosacea environment; significant increase in cell proliferation in a simulated pathological environment; (B) Western blot detection of protein expression changes in TLR2 and S100A9 induced by LL‐37; (C) RT‐qPCR detection of expression changes in typical cytokines IL6, OSM, and TNF‐α induced by LL‐37; (D) RT‐qPCR detection of knockdown efficiency of different shRNA sequences; (E) ELISA detection of secretion levels of cytokines IL6, OSM, and TNF‐α in different treatment groups; (F) scratch assay to assess cell migration in different treatment groups; (G) Transwell assay to assess cell migration in different treatment groups; (H) CCK‐8 assay to assess cell proliferation in the cell groups. The data is presented as mean ± SD, **p < 0.01, ***p < 0.001. Cell experiments were replicated three times.

To confirm the accuracy and reliability of the aforementioned bioinformatics analysis results, we employed an in vitro model using LL‐37 treatment to simulate rosacea. The experimental results demonstrated significant changes in the biological functions and expression of key factors in HaCaT cells under the simulated rosacea environment. First, compared to the control group (NS group), the mRNA and protein levels of TLR2 and S100A9 in the LL‐37 treatment group were markedly elevated in HaCaT cells (Figure 4A,B). Additionally, these cells exhibited enhanced reactivity to inflammatory stimuli. In comparison to the NS group, the LL‐37 treatment group showed a significant increase in the expression levels of IL6, OSM, and TNF‐α in HaCaT cells, confirming the success of our model construction (Figure 4C).

To elucidate the pivotal roles of TLR2 and S100A9 in the pathogenesis of rosacea, we established knockdown cell lines for TLR2 and S100A9. Initially, we assessed the knockdown efficiency of sh‐TLR2 #1 and sh‐S100A9 #1 sequences through RT‐qPCR experiments (Figure 4D). The ELISA results revealed a significant increase in IL6, OSM, and TNF‐α secretion by HaCaT cells in the LL‐37 + shNC group compared to the NS group. Moreover, when compared to the LL‐37 + shNC group, HaCaT cells treated with LL‐37 + shTLR2 exhibited a notable decrease in the secretion of IL6, OSM, and TNF‐α. Similarly, HaCaT cells treated with LL‐37 + shS100A9 displayed a significant reduction in the secretion of IL6, OSM, and TNF‐α when compared to the LL‐37 + shNC group (Figure 4E). Thus, TLR2 and S100A9 play important roles in the inflammatory reactions and immune regulation of rosacea.

Cell migration abilities were assessed through scratch and Transwell assays. The results indicated a significant enhancement in the migration ability of HaCaT cells in the LL‐37 + shNC group compared to the NS group. In contrast, HaCaT cells treated with LL‐37 + shTLR2 exhibited a notable decrease in cell migration ability compared to the LL‐37 + shNC group. Similarly, HaCaT cells treated with LL‐37 + shS100A9 displayed a significant reduction in cell migration ability compared to the LL‐37 + shNC group (Figure 4F,G). Moreover, CCK‐8 assay results demonstrated a significant acceleration in the proliferation of HaCaT cells in the LL‐37 + shNC group compared to the NS group. Conversely, HaCaT cells treated with LL‐37 + shTLR2 exhibited a notable deceleration in cell proliferation compared to the LL‐37 + shNC group. Similarly, HaCaT cells treated with LL‐37 + shS100A9 displayed a significant reduction in cell proliferation compared to the LL‐37 + shNC group (Figure 4H). In summary, keratinocytes demonstrate enhanced proliferation and migration abilities in a rosacea environment. However, this phenomenon can be reversed by knockdown of TLR2 and S100A9. Therefore, TLR2 and S100A9 may be associated with the observed skin thickening and accelerated damage repair processes in rosacea.

### 3.5 High Expression of TLR2 and S100A9 in Skin Tissues of Rosacea Patients

**FIGURE 5:**
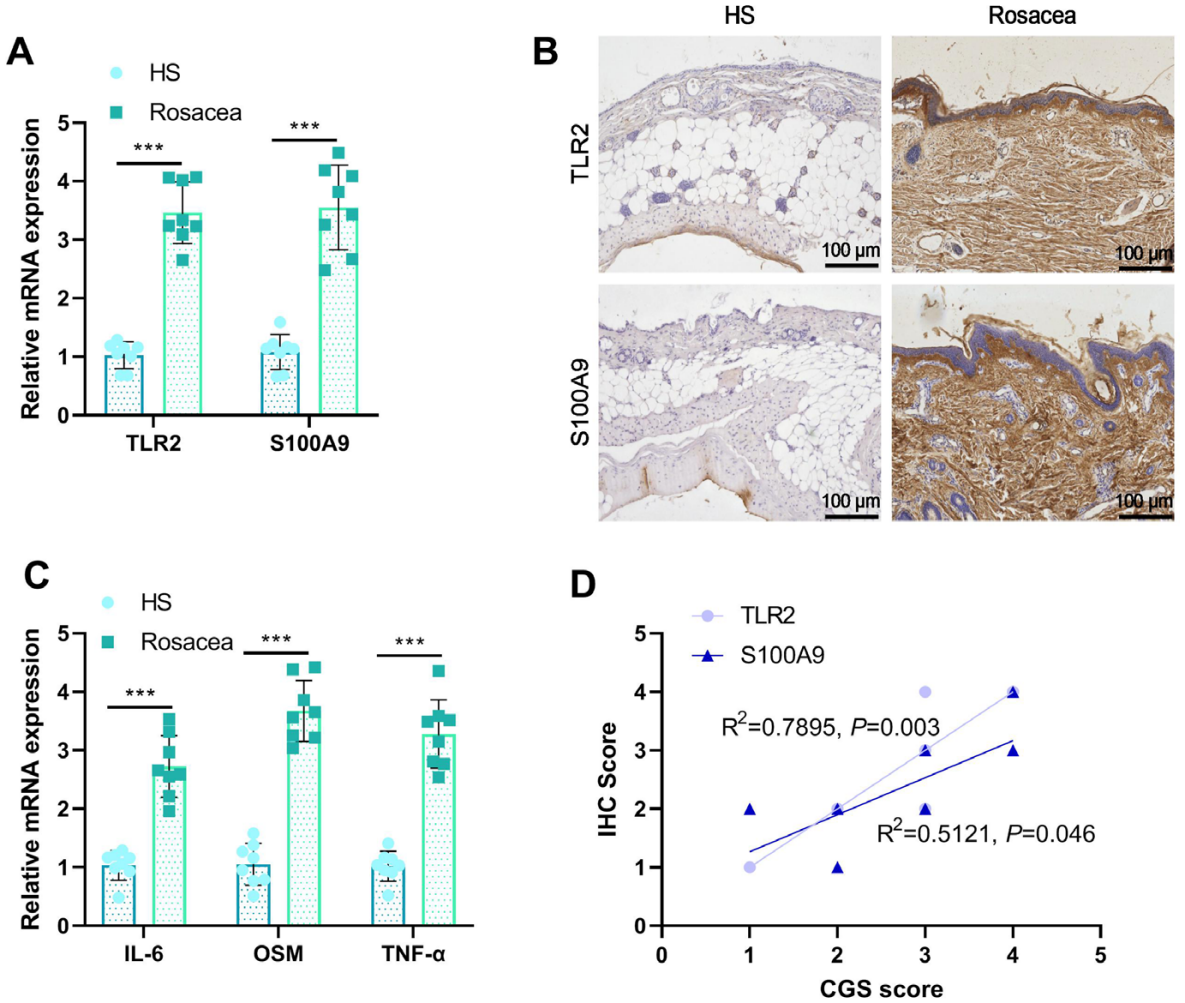
Expression of TLR2 and S100A9 in rosacea patients and their correlation with disease severity. (A) RT‐qPCR detection of TLR2 and S100A9 mRNA expression levels in skin tissue of healthy control group (HS) and rosacea patient group; (B) immunohistochemical staining showing TLR2 and S100A9 protein expression in the skin tissue of HS and rosacea patient groups (100 μm); (C) RT‐qPCR detection of mRNA expression levels of inflammatory factors IL‐6, OSM, and TNF‐α in skin tissue; (D) correlation analysis between IHC scores of TLR2 and S100A9 and CGS scores. ***p < 0.001, HS: *n* = 8, Rosacea: *n* = 8.

To further validate the findings from in vitro cell experiments, we collected facial skin tissue samples from clinical rosacea patients and healthy controls. TLR2 and S100A9 expression levels were first assessed by RT‐qPCR and immunohistochemistry. Compared with the healthy control group, the rosacea patient group showed significantly increased mRNA and protein levels of TLR2 and S100A9 (Figure 5A,B). In addition, RT‐qPCR analysis of inflammatory factors in skin tissue revealed that the expression levels of IL‐6, OSM, and TNF‐α were significantly higher in rosacea patients than in the healthy control group (Figure 5C). Subsequently, we analyzed the correlation between the expression levels of TLR2 and S100A9 and disease severity. The results showed a positive correlation between the expression levels of TLR2 and S100A9 and the CGS score (Figure 5D). These findings indicate that TLR2 and S100A9 are highly expressed in the skin tissues of rosacea patients and are associated with disease severity.

## 4 Discussion

Rosacea is a common chronic skin disease with unclear pathogenic mechanisms [8, 31, 32]. The objective of this study is to explore the molecular mechanisms of rosacea and the effects of drug response through the application of transcriptomics and in vitro experiments. We analyzed the gene expression patterns of rosacea patients using high‐throughput RNA sequencing and further validated them in vitro experiments [33]. Through these experiments, we aim to better understand the pathogenic mechanisms of rosacea and provide important information for the development of therapeutic strategies.

Compared to previous studies, our findings suggest a significant increase in the expression levels of genes associated with inflammatory reactions in rosacea patients [7, 8, 9]. Specifically, the expression of FLT1 and TLR2 genes is significantly elevated in rosacea patients [34]. These results are consistent with previous research and further support the crucial role of inflammatory reactions in the pathogenesis of rosacea [35]. The sustained activation of inflammatory reactions may contribute to the development of skin lesions and further exacerbate the expression of genes related to inflammatory reactions [36, 37].

Our study demonstrates that HaCaT cells exhibit enhanced proliferation and migration abilities under conditions simulating a rosacea environment. This finding is consistent with previous observations of behavioral changes in HaCaT cells in previous studies. The increased proliferation and migration of HaCaT cells may be associated with the upregulation of genes related to inflammatory reactions [38, 39]. These results further support the crucial role of inflammatory reactions in the pathological progression of rosacea [40].

Our findings also reveal a significant upregulation of TLR2 and S100A9 expression in rosacea patients, which is consistent with previous research on TLR2 and S100A9 [41, 42, 43]. The upregulation of TLR2 and S100A9 may promote the pathogenesis of rosacea and inflammatory reaction [44, 45]. TLR2 is involved in the regulation of inflammatory reactions through the activation of the inflammatory pathway, while S100A9 serves as an inflammatory mediator involved in the spread and maintenance of inflammation [45].

**FIGURE 6:**
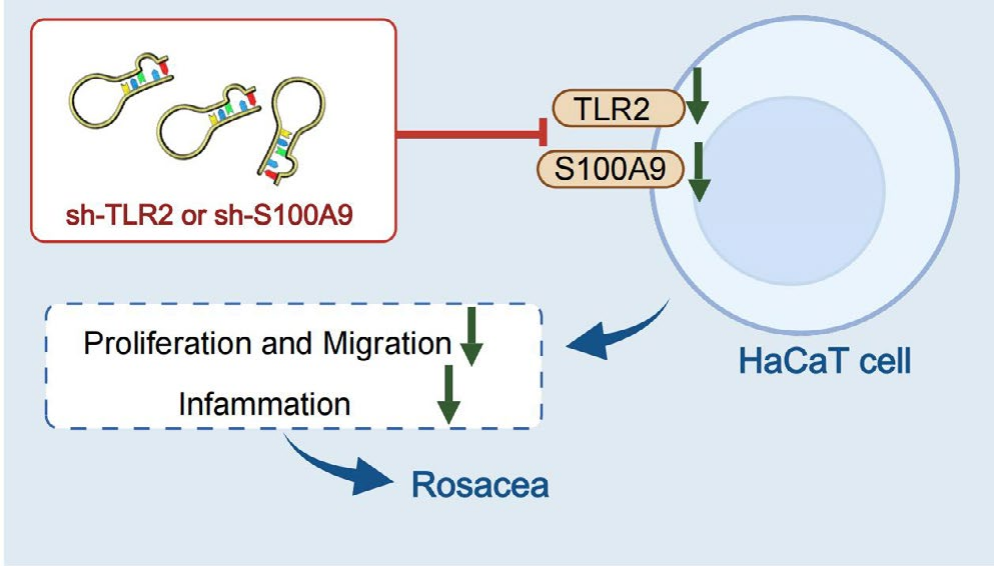
Molecular pathological characteristics of rosacea and individualized treatment strategies.

In summary, this study employed transcriptomics and in vitro experiments to investigate the molecular pathological features of rosacea and identified potential therapeutic approaches. It was found that rosacea is closely associated with a chronic inflammatory state, particularly with significantly elevated expression of genes such as IL6, OSM, and TNF‐α. Rosacea also displayed a close relationship with genes involved in cell proliferation and migration, as evidenced by increased expression of TLR2 and S100A9 (Figure 6). These findings not only enhance our understanding of the pathophysiology of rosacea but also provide a scientific basis for developing treatment strategies targeting specific types of rosacea.

The scientific significance of this study lies in its comprehensive exploration of the molecular mechanisms and drug responses in rosacea through transcriptomics analysis and in vitro experiments. Analysis of the gene expression patterns in rosacea patients revealed elevated levels of genes related to inflammatory reactions, leading to new insights into the pathogenesis of rosacea. In in vitro experiments, the study found that TLR2 and S100A9 protein levels increased in HaCaT cells under conditions simulating rosacea, and the cytokines IL6, OSM, and TNF‐α were significantly elevated, implying their potential promoting roles in the progression of rosacea. Therefore, this study provides important scientific evidence for a deeper understanding and treatment of rosacea.

However, there are still some limitations to this study. First, the study only involved a cell line model, which limits the generalizability of the results, and it is possible that the results may differ from other animal models. In addition, more clinical data and human studies are needed to validate the reliability and applicability of these results.

Future research can further explore the molecular mechanisms and drug responses in rosacea. More key molecules can be studied at the genomic and proteomic levels to reveal the pathological mechanisms of rosacea. Additionally, in vivo experiments and clinical research can further validate the reliability of the research results and further optimize and improve existing treatment strategies. Personalized therapy can also be a future research direction, with the development of treatment strategies targeting specific molecular targets based on a deeper understanding of the pathogenesis of rosacea, providing more precise, safe, and effective treatment options. In conclusion, these prospects will contribute to the advancement of rosacea research and provide important guidance and decision‐making basis for clinical practice.

## Author Contributions

Luzhu Chen and Juan Wang contributed to the conception and design of the study. Luzhu Chen performed the transcriptomic analysis, in vitro experiments, and data interpretation. Juan Wang supervised the project, provided critical revisions, and ensured the accuracy of the methodology. Both authors contributed to drafting and revising the manuscript and approved the final version for publication.

## Conflicts of Interest

The authors declare no conflicts of interest.

## Supporting information


**Figure S1:** Flowchart of in vitro cell experiments.

Table S1:

Table S2:

Table S3:

## Data Availability

All data can be provided as needed.
