# Correction to “Unveiling the Molecular Mechanisms of Rosacea: Insights From Transcriptomics and In Vitro Experiments”

**DOI:** 10.1111/jocd.70165

**Published:** 2025-09-25

L. Chen and J. Wang, “Unveiling the Molecular Mechanisms of Rosacea: Insights From Transcriptomics and In Vitro Experiments,” Journal of Cosmetic Dermatology 24 (2025): e16753, 10.1111/jocd.16753.

In the funding acknowledgments, the grant number is incorrect. It should be revised from “This work was supported by National Natural Science Foundation of China (No. 81873347)” to “This work was supported by National Natural Science Foundation of China (No. 82372081).”

We apologize for this error.

