# Supplementary figures and images for: Unveiling the Molecular Mechanisms of Rosacea: Insights From Transcriptomics and In Vitro Experiments
Source: J Cosmet Dermatol. 2025 Jan 16;24(1):e16753. doi: 10.1111/jocd.16753 (PMC11739675; doi:10.1111/jocd.16753)

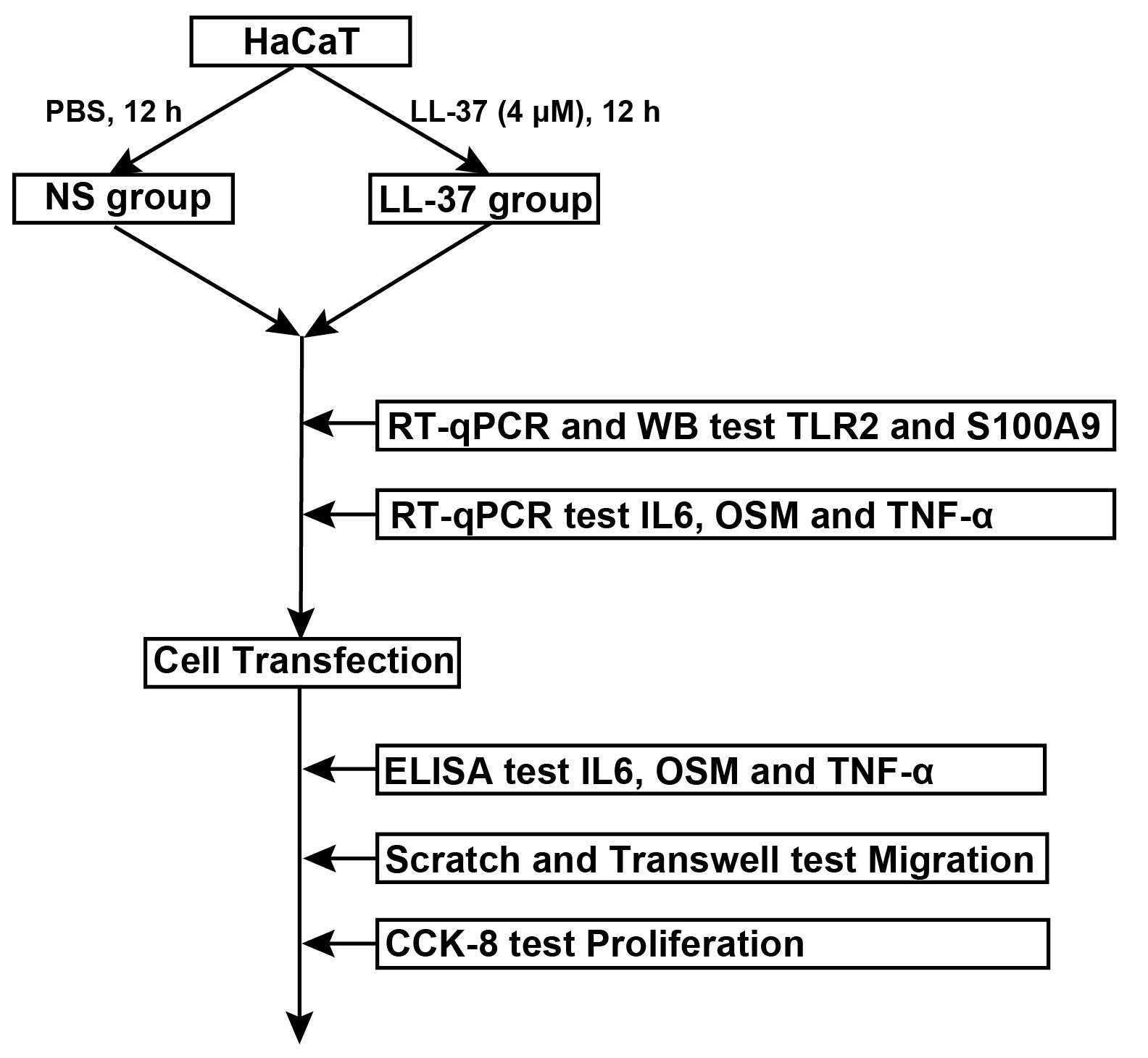

Supplement: Supplementary file 1 — Figure S1: Flowchart of in vitro cell experiments. [file JOCD-24-e16753-s004.jpg]
