# Supplementary material for: Unveiling the Molecular Mechanisms of Rosacea: Insights From Transcriptomics and In Vitro Experiments
Source: J Cosmet Dermatol. 2025 Jan 16;24(1):e16753. doi: 10.1111/jocd.16753 (PMC11739675; doi:10.1111/jocd.16753)
Supplement: Supplementary file 3 — Table S2: [file JOCD-24-e16753-s003.docx]

**Table S2. qRT-RCR primer sequence**

| **Target** | **Sequence (5'-3')** |
| --- | --- |
| TLR2(human) | F: GGCGTTCTCTCAGGTGACTG |
|  | R: CCCTGTCTTCCTGCCTTCAC |
| S100A9(human) | F: GCAGCTGGAACGCAACATAG |
|  | R: CCACTGTGATCTTGGCCACT |
| IL6(human) | F: CCTTCGGTCCAGTTGCCTTCT |
|  | R: TCTGAGGTGCCCATGCTACA |
| TNF-α(human) | F: GACAAGCCTGTAGCCCATGT |
|  | R: GGAGGTTGACCTTGGTCTGG |
| OSM(human) | F: GAGCAGCTGACAAGGTCTGG |
|  | R: TCAGCCGTGTCTGAGTTGTC |
| GAPDH(human) | F: GAGAAGGCTGGGGCTCATTT |
|  | R: AGTGATGGCATGGACTGTGG |
| IL6(mouse) | F: GCCTTCTTGGGACTGATGCT |
|  | R: TGTGACTCCAGCTTATCTCTTGG |
| TNF-α(mouse) | F: ACCCTCACACTCACAAACCA |
|  | R: ACCCTGAGCCATAATCCCCT |
| OSM(mouse) | F: AAGGAACACTGATCTGGGCG |
|  | R: TTGCACCACAGGTTCCCATT |
| GAPDH(mouse) | F: GCCTCCTCCAATTCAACCCT |
|  | R: CTCGTGGTTCACACCCATCA |
