# Supplementary material for: Unveiling the Molecular Mechanisms of Rosacea: Insights From Transcriptomics and In Vitro Experiments
Source: J Cosmet Dermatol. 2025 Jan 16;24(1):e16753. doi: 10.1111/jocd.16753 (PMC11739675; doi:10.1111/jocd.16753)
Supplement: Supplementary file 4 — Table S3: [file JOCD-24-e16753-s001.docx]

**Table S3. GSE65914 sample information**

| **Accession** | **Source Name** | **Group** |
| --- | --- | --- |
| GSM1611035 | Human skin | Healthy volunteer |
| GSM1611036 | Human skin | Healthy volunteer |
| GSM1611037 | Human skin | Healthy volunteer |
| GSM1611038 | Human skin | Healthy volunteer |
| GSM1611039 | Human skin | Healthy volunteer |
| GSM1611040 | Human skin | Healthy volunteer |
| GSM1611041 | Human skin | Healthy volunteer |
| GSM1611042 | Human skin | Healthy volunteer |
| GSM1611043 | Human skin | Healthy volunteer |
| GSM1611044 | Human skin | Healthy volunteer |
| GSM1611045 | Human skin | Healthy volunteer |
| GSM1611046 | Human skin | Healthy volunteer |
| GSM1611047 | Human skin | Healthy volunteer |
| GSM1611048 | Human skin | Healthy volunteer |
| GSM1611049 | Human skin | Healthy volunteer |
| GSM1611050 | Human skin | Healthy volunteer |
| GSM1611051 | Human skin | Healthy volunteer |
| GSM1611052 | Human skin | Healthy volunteer |
| GSM1611053 | Human skin | Healthy volunteer |
| GSM1611054 | Human skin | Healthy volunteer |
| GSM1611055 | Human skin | erythematotelangiectatic rosacea (ETR) |
| GSM1611056 | Human skin | erythematotelangiectatic rosacea (ETR) |
| GSM1611057 | Human skin | erythematotelangiectatic rosacea (ETR) |
| GSM1611058 | Human skin | erythematotelangiectatic rosacea (ETR) |
| GSM1611059 | Human skin | erythematotelangiectatic rosacea (ETR) |
| GSM1611060 | Human skin | erythematotelangiectatic rosacea (ETR) |
| GSM1611061 | Human skin | erythematotelangiectatic rosacea (ETR) |
| GSM1611062 | Human skin | erythematotelangiectatic rosacea (ETR) |
| GSM1611063 | Human skin | erythematotelangiectatic rosacea (ETR) |
| GSM1611064 | Human skin | erythematotelangiectatic rosacea (ETR) |
| GSM1611065 | Human skin | erythematotelangiectatic rosacea (ETR) |
| GSM1611066 | Human skin | erythematotelangiectatic rosacea (ETR) |
| GSM1611067 | Human skin | erythematotelangiectatic rosacea (ETR) |
| GSM1611068 | Human skin | erythematotelangiectatic rosacea (ETR) |
| GSM1611069 | Human skin | papulopustular rosacea (PPR) |
| GSM1611070 | Human skin | papulopustular rosacea (PPR) |
| GSM1611071 | Human skin | papulopustular rosacea (PPR) |
| GSM1611072 | Human skin | papulopustular rosacea (PPR) |
| GSM1611073 | Human skin | papulopustular rosacea (PPR) |
| GSM1611074 | Human skin | papulopustular rosacea (PPR) |
| GSM1611075 | Human skin | papulopustular rosacea (PPR) |
| GSM1611076 | Human skin | papulopustular rosacea (PPR) |
| GSM1611077 | Human skin | papulopustular rosacea (PPR) |
| GSM1611078 | Human skin | papulopustular rosacea (PPR) |
| GSM1611079 | Human skin | papulopustular rosacea (PPR) |
| GSM1611080 | Human skin | papulopustular rosacea (PPR) |
| GSM1611081 | Human skin | phymatous rosacea (PhR) |
| GSM1611082 | Human skin | phymatous rosacea (PhR) |
| GSM1611083 | Human skin | phymatous rosacea (PhR) |
| GSM1611084 | Human skin | phymatous rosacea (PhR) |
| GSM1611085 | Human skin | phymatous rosacea (PhR) |
| GSM1611086 | Human skin | phymatous rosacea (PhR) |
| GSM1611087 | Human skin | phymatous rosacea (PhR) |
| GSM1611088 | Human skin | phymatous rosacea (PhR) |
| GSM1611089 | Human skin | phymatous rosacea (PhR) |
| GSM1611090 | Human skin | phymatous rosacea (PhR) |
| GSM1611091 | Human skin | phymatous rosacea (PhR) |
| GSM1611092 | Human skin | phymatous rosacea (PhR) |

**Table S2. qRT-RCR primer sequence**

| **Target** | **Sequence (5'-3')** |
| --- | --- |
| TLR2(human) | F: GGCGTTCTCTCAGGTGACTG |
|  | R: CCCTGTCTTCCTGCCTTCAC |
| S100A9(human) | F: GCAGCTGGAACGCAACATAG |
|  | R: CCACTGTGATCTTGGCCACT |
| IL6(human) | F: CCTTCGGTCCAGTTGCCTTCT |
|  | R: TCTGAGGTGCCCATGCTACA |
| TNF-α(human) | F: GACAAGCCTGTAGCCCATGT |
|  | R: GGAGGTTGACCTTGGTCTGG |
| OSM(human) | F: GAGCAGCTGACAAGGTCTGG |
|  | R: TCAGCCGTGTCTGAGTTGTC |
| GAPDH(human) | F: GAGAAGGCTGGGGCTCATTT |
|  | R: AGTGATGGCATGGACTGTGG |
| IL6(mouse) | F: GCCTTCTTGGGACTGATGCT |
|  | R: TGTGACTCCAGCTTATCTCTTGG |
| TNF-α(mouse) | F: ACCCTCACACTCACAAACCA |
|  | R: ACCCTGAGCCATAATCCCCT |
| OSM(mouse) | F: AAGGAACACTGATCTGGGCG |
|  | R: TTGCACCACAGGTTCCCATT |
| GAPDH(mouse) | F: GCCTCCTCCAATTCAACCCT |
|  | R: CTCGTGGTTCACACCCATCA |

**Table S3. Information of patients and healthy individuals skin samples.**

| **Sample ID** | **Sample classification** | **Age/Gender** | **CGS score** |
| --- | --- | --- | --- |
| 1 | HS | 35/Female | - |
| 2 | HS | 33/Female | - |
| 3 | HS | 43/Female | - |
| 4 | HS | 45/Female | - |
| 5 | HS | 28/Female | - |
| 6 | HS | 26/Female | - |
| 7 | HS | 42/Female | - |
| 8 | HS | 29/Female | - |
| 9 | Rosacea | 30/Female | 3 |
| 10 | Rosacea | 28/Female | 2 |
| 11 | Rosacea | 35/Female | 3 |
| 12 | Rosacea | 40/Female | 3 |
| 13 | Rosacea | 27/Female | 4 |
| 14 | Rosacea | 41/Female | 4 |
| 15 | Rosacea | 39/Female | 2 |
| 16 | Rosacea | 40/Female | 1 |
